# Central correlates of placebo effects in nausea differ between men and women

**DOI:** 10.1002/brb3.2685

**Published:** 2022-07-10

**Authors:** Anja Haile, Mallissa Watts, Simone Aichner, Franziska Stahlberg, Verena Hoffmann, Matthias H. Tschoep, Karin Meissner

**Affiliations:** ^1^ Institute of Medical Psychology, Faculty of Medicine LMU Munich Munich Germany; ^2^ Helmholtz Diabetes Center and German Center for Diabetes Research Helmholtz Zentrum München Neuherberg Germany; ^3^ Division of Metabolic Diseases, Department of Medicine Technical University of Munich Munich Germany; ^4^ Division of Health Promotion Coburg University of Applied Sciences & Arts Coburg Germany

**Keywords:** cortisol, electroencephalography, motion sickness, nausea, placebo effect

## Abstract

**Introduction:**

Despite growing evidence validating placebo effects in nausea, little is known about the underlying cortical mechanisms in women and men. Therefore, the present study examined sex differences and electroencephalography (EEG) characteristics of the placebo effect on nausea.

**Methods:**

On 2 consecutive days, 90 healthy subjects (45 females) were exposed to a nauseating visual stimulus. Nausea was continuously rated on an 11‐point numeric rating scale, and 32 EEG channels were recorded. On day 2, subjects were randomly allocated to either placebo treatment or no treatment: the placebo group received sham acupuncture, whereas the control group did not receive any intervention.

**Results:**

In contrast to the control group, both sexes in the placebo group showed reduced signs for anticipatory nausea in the EEG, indexed by increased frontal lobe and anterior cingulate activity. Among women, the improvement in perceived nausea in the placebo group was accompanied by decreased activation in the parietal, frontal, and temporal lobes. In contrast, the placebo‐related improvement of perceived nausea in men was accompanied by increased activation in the limbic and sublobar (insular) lobes.

**Conclusion:**

Activation of the parietal lobe in women during the placebo intervention may reflect altered afferent activity from gastric mechanoreceptors during nausea‐induced tachyarrhythmia, whereas in men, altered interoceptive signals in the insular cortex might play a role. Thus, the results suggest different cerebral mechanisms underlying the placebo effects in men and women, which could have implications for the treatment of nausea.

## Graphical abstract

In the present study, EEG characteristics during anticipated and acute nausea were investigated in an experimental nausea paradigm in 45 men and 45 women. Compared with no treatment, the placebo intervention reduced the expected intensity of nausea inall participants, which was associated with increased ACC and PFC activity. Furthermore, the placebo intervention alleviated acute nausea similarly in both sexes, whereas cortical changes differed: Women showed lower activation in the parietal, frontal, and temporal lobes, while men displayed increased activation in the limbic and insular lobes.

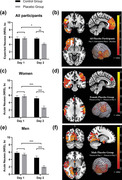

## 1 INTRODUCTION

Nausea is a widespread and highly individual condition that ranges from relatively harmless during motion in approximately 30% of car drivers (Turner & Griffin, 1999) to intolerable in 25% of cancer patients receiving chemotherapy (Morrow et al., 1999) and in 52% of pregnant women (Gadsby et al., 1993). Its signs and symptoms include gastrointestinal (e.g., stomach ache), emotional (e.g., stress), cortical (e.g., changes in electroencephalography [EEG] characteristics), and gastrointestinal (e.g., stomach ache) afflictions (Levine, 2017).

While some medical treatments of nausea lead to the remission of symptoms (e.g., vomiting; Jordan et al., 2007), most medical approaches fail to successfully alleviate nausea (for a review, see Sanger & Andrews, 2006). The mechanisms underlying such failure in reducing symptoms are not fully understood but may involve the missed attempt to explicitly trigger the psychosocial components of nausea, for example, to take away high initial expectations of nausea (Colagiuri & Zachariae, 2010).

A convincing positive expectancy manipulation was also identified as a key component in inducing powerful placebo effects in nausea (Quinn & Colagiuri, 2015). This was shown, for example, in cancer patients treated with radiation therapy; nausea could be effectively reduced following sham acupuncture but only in those subjects who believed that the treatment would effectively prevent nausea (Enblom et al., 2012). In pain and depression, cortical expectation networks activated by expectations about treatment outcome contributed to the beneficial effects of specific medications (Benedetti, 2010; Hunter et al., 2006). In particular, similar brain activity, e.g., in the prefrontal cortex (PFC) and the anterior cingulate cortex (ACC), during the anticipation phase (i.e., before the sham treatment starts, through which the participants anticipate symptom relief), was observed when comparing a placebo group to a group of patients receiving a real medication (Colloca & Benedetti, 2005). Despite growing evidence validating placebo effects in nausea, little is known about the underlying cortical mechanisms. To fill this gap, the present study aimed to reveal the EEG characteristics during anticipatory and acute nausea in an experimental nausea paradigm in men and women.

To this end, we experimentally studied nausea in the context of motion sickness. Motion sickness can be induced by means of a virtual vection drum that works through illusory self‐motion. Vection drums usually consist of black and white stripes moving constantly from left to right (Reason & Brand, 1975). Bodily changes of acute nausea in the context of motion sickness are well studied and have been suggested to act in a network composed of mechanisms within the autonomic nervous system, the central nervous system, and the endocrine system (Farmer et al., 2015; Singh et al., 2016). On the cortical level, acute nausea has been associated with changes in the insular cortex, ACC, orbitofrontal cortex, somatosensory cortex, and PFC (Farmer et al., 2015; Napadow et al., 2013). Specific frequencies of the fast Fourier transformation (FFT) spectrum of the EEG were also shown to be altered during acute nausea. For example, Hu et al. (1999) reported that the percentage of delta power (compared to the whole spectrum of ‘‘0’’ to ‘‘30’’ Hz) in central electrodes C3 and C4 was increased during acute nausea.

Notably, gender does not appear to have a significant influence on the placebo effect on a behavioral level, for example, in pain and depression (Averbuch & Katzper, 2001; Casper et al., 2001; Weimer et al., 2015) but instead plays a larger modulatory role on the cortical and hormonal levels (Colloca et al., 2016; Theysohn et al., 2014). This demonstrates the importance of studying possible gender differences on a cortical level as well.

The purpose of this study was to investigate the cortical mechanisms related to the placebo effect in nausea and to explore possible differences between men and women. Ninety healthy participants were exposed for 20 min to a virtual vection drum on 2 separate days. On the second day, participants were randomly allocated to placebo treatment, that is, sham transcutaneous electrical nerve stimulation (TENS) of a dummy acupuncture point coupled with expectancy manipulation of nausea improvement, or no treatment. The results demonstrated significant placebo effects on nausea and motion sickness (Aichner et al., 2019) as well as on gastric myoelectrical activity in women (Meissner et al., 2020). Here, we present the EEG characteristics and stress measures collected during the study to better understand the central mechanisms of placebo effects in nausea.

We hypothesized that (I) the placebo intervention would alter brain activity in the PFC and ACC during the anticipation phase (i.e., before nausea induction) in comparison to no treatment, (II) the placebo intervention would alleviate changes in nausea‐related EEG activity in the placebo group compared to the control group, and (III) central changes would differ between women and men during sham TENS (Vambheim & Flaten, 2017).

## 2 METHODS

### 2.1 Study design

This randomized controlled, four‐arm, parallel group study was conducted at the Institute of Medical Psychology, Ludwig‐Maximilians‐University (LMU) of Munich, Munich, Germany. Full details of the trial participants, design, and results have been previously published (Aichner et al., 2019; Meissner et al., 2020). In brief, 100 healthy participants were exposed to a virtual vection drum on 2 separate days, referred to as day 1 (control day) and day 2 (intervention day). On day 2, participants were randomly allocated to one of three treatment arms: placebo treatment (i.e., sham TENS of a sham acupuncture point; *n* = 60), active treatment (i.e., TENS of the acupuncture point PC6; *n* = 10), or no treatment (*n* = 30). All groups were stratified by sex (50% women, 50% men). The study was conducted in compliance with the World Medical Association's Declaration of Helsinki (1964 and its later amendments). Informed consent was obtained from all subjects. The study protocol was approved by the ethical review committee of the Medical Faculty at LMU Munich (No. 402−13).

### 2.2 Experimental procedure

**FIGURE 1:**
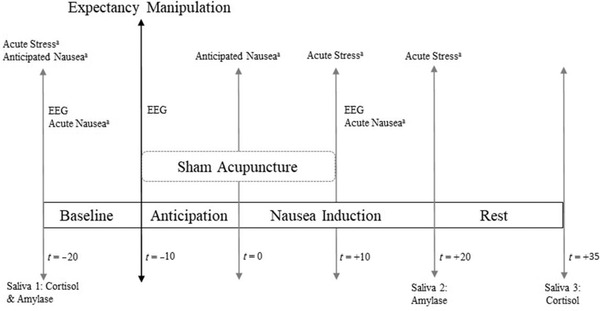
Overview of the study protocol on day 2. Expectancy manipulation and sham acupuncture were only applied in the placebo group. The control group received no intervention. ^a^Numeric rating scale (0–10). Abbreviations: Bc, baseline corrected; EEG, electroencephalography

The experimental procedure is summarized in Figure 1. On both days, the electrodes for psychophysiological assessments (Meissner et al., 2020), including the EEG net, were attached, and an indwelling catheter was fixed at the forearm to allow for repeated blood drawings (results reported in Meissner et al., 2020). Both sessions started with a 10‐min resting period, after which randomization and treatment allocation were performed on day 2. Then, a 10‐min anticipation period took place, followed by a 20‐min period of nausea induction. The experimental sessions included a 15‐min resting period.

### 2.3 Nausea induction

Nausea was induced by standardized visual presentation of alternating black and white stripes with left‐to‐right circular motion at 60 degree/s. This left‐to‐right horizontal translation induces a circular vection sensation wherein subjects experience a false sensation of translating to the left (Napadow et al., 2013). The nauseating stimulus was projected to a semicylindrical and semitransparent screen placed around the volunteer at a distance of 30 cm to the eyes. Such stimulation simulates visual input provided by a rotating optokinetic drum, commonly used to induce vection (illusory self‐motion) and thereby nausea (Levine, 2017). For security reasons, the vection stimulus was stopped if nausea ratings indicated severe nausea (ratings of 9 or 10 on the 11‐point NRS).

### 2.4 Placebo intervention

Placebo and active interventions were implemented by means of a programmable transcutaneous electrical nerve stimulation (TENS) device (Digital EMS/TENS unit SEM 42; Sanitas, Uttenweiler, Germany). For the active intervention, the electrodes were placed around PC6, a validated acupuncture point for the treatment of nausea (Witt et al., 2012), and the TENS program was turned on for 20 min. For the placebo treatment, the electrodes were attached just proximal and distal to a nonacupuncture point at the ulnar side of the forearm generally accepted to represent a dummy point in the context of acupuncture research (Witt et al., 2012).

Two types of placebo stimulation were applied: 30 participants (15 males, 15 females) received subtle stimulation at a very low intensity by turning on the massage program of the TENS device, while 30 participants (15 males, 15 females) received no electric stimulation at all. Because the two placebo groups with and without electrotactile stimulation showed comparable effects (Aichner et al., 2019), we combined the participants of both groups into one large placebo group (*n* = 60) to enhance statistical power. Sensitivity analyses for the EEG parameters were performed to ensure that the two placebo groups were comparable on a cortical level as well (Section 3).

### 2.5 Blinding and randomization

The active treatment group (data not analyzed) was included to allow for the blinded administration of the placebo intervention, a common approach in placebo studies (Benedetti et al., 2003). The no‐treatment group served to control the placebo effect for naturally occurring changes from day 1 to day 2. Computer‐assisted randomization was performed by a person not involved in the experiments who prepared sequentially numbered, sealed, and opaque randomization envelopes. The experimenters administering the placebo treatments were not aware of treatment allocation. Study interventions were performed in a single‐blind design, while participants in the no‐treatment control group remained necessarily unblinded. Previous analyses showed successful blinding of the placebo interventions (Aichner et al., 2019).

### 2.6 Electroencephalography recordings

EEG was recorded using the 32‐channel ActiveTwo system (BioSemi) sampled at 2 kHz. The offset signal was controlled individually for each channel and kept below 20 mV. The EEG data were processed offline using Brain Vision Analyzer 2.0. Sinc‐Interpolation was used to downsample the EEG data to a rate of 256 Hz. A low cutoff of 0.5 Hz and a high cutoff of 50 Hz with a notch filter of 50 Hz were applied. Gross artifacts were removed, and EEG data were rereferenced to the mastoids. Next, ocular ICA was run to remove blink artifacts. Bad individual channels were spatially weighted and linearly interpolated. To exclude any remaining artifacts, a final semiautomatic artifact rejection was applied. Additionally, voltage levels at the 32 electrodes were replaced by valid head coordinates through the current source density (order of splines Enblom et al., 2012) (maximum degree of Legendre polynomials Reason & Brand, 1975). Then, each 10‐min period was segmented into 1‐s segments and subjected to FFT.

### 2.7 Delta power

Mean values of the following frequency bands were analyzed in SPSS: δ, 0.5–4 Hz, θ, 4.1–8 Hz; α, 8.1–13 Hz; β I, 13.1–20 Hz; β II, 20.1–30 Hz; and total EEG (0.5–30 Hz) for both the baseline period and during nausea exposure (frequency bands adapted from Hu et al., 1999).

Mean delta values of spectral power were extracted from electrodes C3 and C4 during baseline and acute nausea on both days. The increase in delta power during acute nausea was computed as the delta percentage of total power during nausea minus the delta percentage of total power during the baseline delta. The percentage of delta power was defined as spectral delta power in relation to total spectral power (total EEG power, 0.5–30 Hz; see Hu et al., 1999).

### 2.8 Exact low resolution electromagnetic tomography

The raw EEG data were preprocessed offline in Brain Vision Analyzer 2.0 (see 2.6) and segmented into 1‐min sequences in exact low resolution electromagnetic tomography (eLORETA) (Pascual‐Marqui, 2002, 2007). Voxelwise *t*‐tests were performed by comparing the relevant conditions. All *p*‐values were corrected for multiple comparisons using the nonparametric permutation approach (Nichols & Holmes, 2002) implemented in eLORETA. Significant changes in the placebo group during anticipatory and acute nausea in the different frequency oscillations (δ, 0.5–4 Hz, θ, 4.1–8 Hz; α, 8.1–13 Hz; β I, 13.1–20 Hz; β II, 20.1–30 Hz) were defined at *p* ≤ .05.

### 2.9 Cortisol and amylase

Saliva samples of cortisol and alpha‐amylase were collected using a cotton swab on which participants chewed on for at least 60 s before storing it in a tube. After each session, saliva samples were centrifuged at 2000 rpm at 4°C and 2 × 300 μl per sample and stored at −20°C until analysis.

Salivary amylase and cortisol levels were determined using the ‘‘cortisol saliva assay’’ and ‘‘alpha amylase saliva assay’’ kits from IBL International GmbH (catalog number of cortisol kit: RE52611 and amylase: RE80111). The values of amylase and cortisol levels were logarithmized (ln) and corrected for the levels measured at baseline by computing for amylase: ‘‘level at acute nausea minus level at baseline’’ and for cortisol: ‘‘level at the end of the session minus level at baseline.’’

### 2.10 Statistical analysis

Sample size calculation was performed for assumed group differences in behavioral nausea, as reported earlier (Aichner et al., 2019). Levene's test was used to ensure equal variances between groups. Mixed 2 × 2 × 2 analyses of variance (ANOVAs) were conducted for the behavioral as well as for the EEG outcomes to examine the magnitude of nausea induction and the placebo effect on nausea, the latter being defined as the decrease in the magnitude of nausea sensation from day 1 to day 2 in the placebo group compared with the control group. The between‐subject factors were defined as *group* (no treatment versus sham acupuncture point stimulation) and *sex* (male versus female), and the within‐subject factor was defined as *day* (control day versus intervention day). Univariate ANOVAs and post hoc *t*‐tests were conducted following significant ANOVA results. To identify brain regions reflecting changes in activity during the anticipatory period, voxelwise paired *t*‐tests were conducted, comparing the EEG recordings on day 2 during baseline to the recordings during the anticipation period separately for the control and the placebo groups. To identify brain regions reflecting placebo‐induced changes in nausea‐related activity, voxelwise paired *t*‐tests were performed, comparing the EEG characteristics during nausea induction on day 1 and day 2 separately for the control and placebo groups. For all statistical tests, a *p*‐value of ≤ .05 (two‐tailed) was considered statistically significant.

## 3 RESULTS

### 3.1 Sample

The placebo and no‐treatment control groups were comparable with regard to sociodemographic, physical, and psychological characteristics at baseline (Table 1).

**TABLE 1:** Overview of demographic and psychological characteristics at baseline

	Control group (*n* = 30)		Placebo group (n = 60)		
	Mean	SD	Mean	SD	*p*‐Value
Age (years)	23.50	2.70	23.45	3.42	.94
Education (years)	16.16	3.30	16.67	2.34	.40
BMI (kg/m^2^)	22.31	2.75	21.61	2.10	.19
MSSQ	141.55	42.92	135.57	36.48	.51
HADS‐D	1.41	1.57	1.76	1.64	.34
HADS‐A	3.96	2.65	4.00	2.19	.95
STAI‐trait	38.75	6.39	37.78	6.49	.51
STAI‐state day 1	34.70	4.87	35.79	8.33	.51
STAI‐state day 2	35.51	8.96	34.88	8.14	.74

*Note*: Entries show the mean and standard deviation (SD) and *p*‐values (one‐way ANOVA). Abbreviations: BMI, body mass index; MSSQ, motion sickness susceptibility questionnaire; HADS‐D, hospital depression scale; HADS‐A, hospital anxiety scale: STAI, state‐trait anxiety inventory.

### 3.2 Anticipatory changes: Behavioral and eLORETA

**FIGURE 2:**
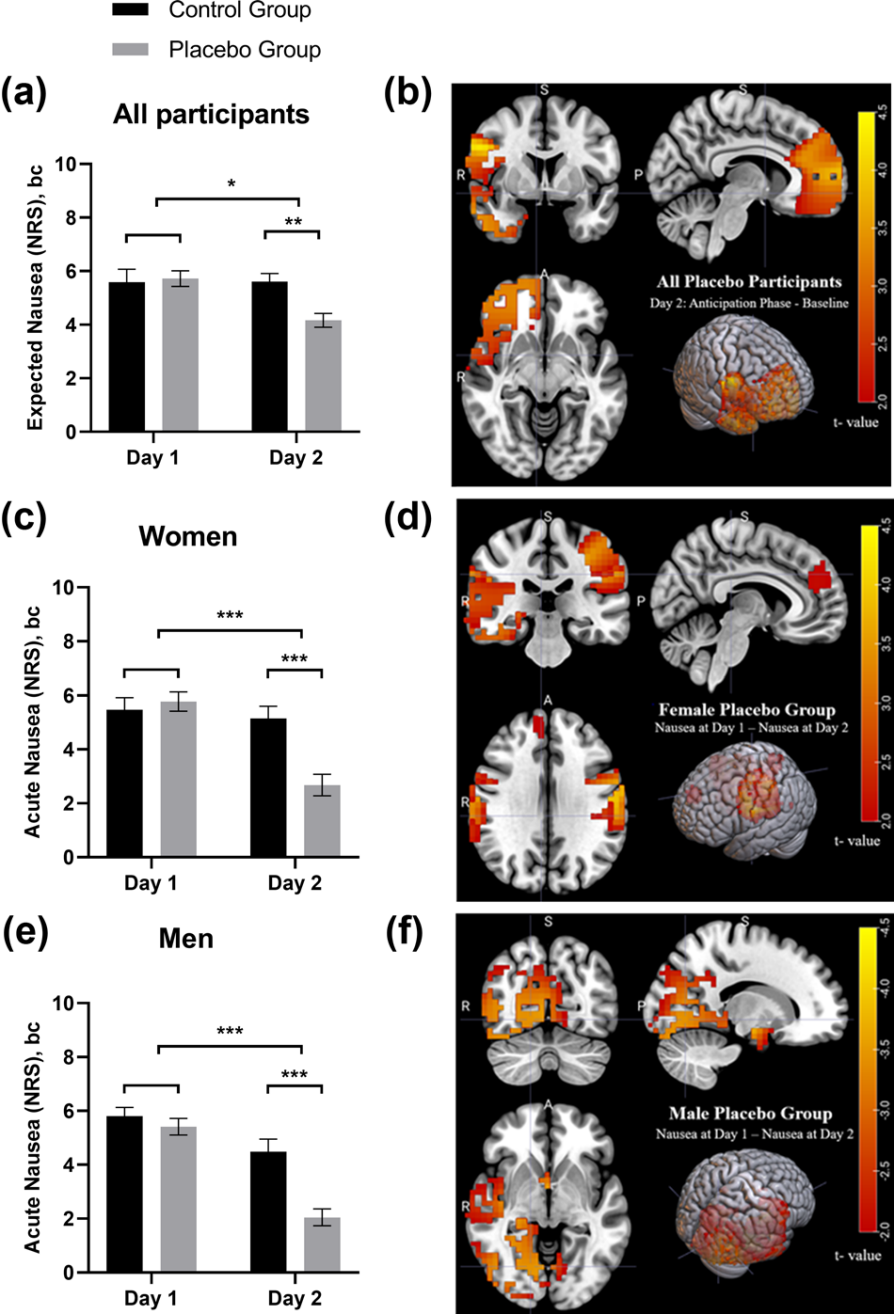
Behavioral and cortical changes before and during nausea. (a) Numeric rating scale (NRS) of expected nausea by group and day. (b) Comparison of the anticipation phase following expectancy manipulation (mean over −10 to 0 min) and baseline (mean over −20 to −10 min) at day 2 for the placebo group, including both sexes. The color bar represents the *t* statistic with *t* ≥ 3.63 indicating *p* ≤ .05. (c) NRS ratings of acute nausea by group and day in female participants. (d) Comparison of acute nausea at day 1 (mean over +10 to +20 min) and acute nausea at day 2 (mean over +10 to +20 min) in the female placebo group. The color bar represents the t‐statistic, with *t* ≥ 3.98 indicating *p* ≤ .05. (e) NRS ratings of acute nausea by group and day in male participants. (f) Comparison of acute nausea at day 1 (mean over +10 to +20 min) and acute nausea at day 2 (mean over +10 to +20 min) in the male placebo group. The color bar represents the t‐statistic, with *t* ≤ −3.57 indicating *p* ≤ .05. Source: Montreal Neurological Institute coordinate system (MNI): R=right, A=Anterior, P=Posterior, S=Superior. Error bars indicate standard error. *p ≤ .05, **p ≤ .01, ***p ≤ .001

A main effect of *day* (control versus intervention day) emerged for expected nausea (*F*(1, 80) = 6.13, *p *= .02) as well as a two‐way interaction between *day* × *group* (*F*(1, 80) = 5.87, *p* = .02). As hypothesized, the interaction was driven by lower levels of expected nausea in the placebo group (*M* = 4.2, SE = 1.9 SE) compared with the control group (*M* = 5.6, SE = 1.6) on day 2 (Bonferroni‐corrected post hoc test, *p* = .002; Figure 2a). This indicates that the expectancy manipulation elicited the intended effect on a behavioral level. Neither a two‐way interaction of *day* × *sex* nor a three‐way interaction between *day* × *group* × *sex* emerged (*p* ≥ .05).

In line with the main hypothesis on the source localization analysis, eLORETA showed an expectation‐related increase from baseline on day 2 during the anticipatory period (from −10 min to 0, the time period following expectancy manipulation) in delta (0.5–4 Hz) activation arising from peak voxels in the frontal lobe and anterior cingulate cortex (extreme *p* ≤ .001, *t* ≥ 3.63, Figure 2b, Table 2), as well as increased theta (4.1–8) oscillations arising from peak voxels in the frontal lobe. In contrast, no significant differences between the recordings during baseline and after randomization on day 2 were found in the control group (*p* ≥ .05). When stratified by sex, no significant changes from baseline in the placebo group during the anticipatory phase emerged (*p* ≥ .05). Sensitivity analyses were performed to test whether the anticipatory EEG changes were related to the presence or absence of electrotactile stimulation of the TENS device in the two original placebo groups. In both the placebo groups with and without electrotactile stimulation, eLORETA showed activation from peak voxels in the frontal lobe in the delta spectrum (*p*‐values ≤ .05).

**TABLE 2:** Coordinates for brain areas showing significant differences (a) during the anticipation phase following expectancy manipulation on day 2 in the placebo group compared to baseline on day 2; in (b) women and (c) in men during acute nausea following the placebo intervention on day 2 compared to acute nausea on day 1

		Peak voxel	*t*‐Value
Frequency	Lobe, structure, brodmann area	(X, Y, Z)	(two‐tailed)
(a) Anticipation phase (*n* = 60), *t*‐values ≥ 3.63 indicate *p*‐values ≤ .05			
Delta, theta	Frontal lobe, 6, 9, 10	55, 0, 30	4.30
Delta	Anterior cingulate, 32	10, 40, 15	3.83
(b) Placebo intervention: Female group (*n* = 30), *t*‐values ≥ 3.98 indicate *p*‐values ≤ .05			
Alpha	Parietal lobe, postcentral gyrus, 1, 3	−65, −20, 35	4.04
Alpha	Frontal lobe, precentral gyrus, 4	10, 40, 15	4.04
Beta I	Temporal lobe, middle temporal gyrus, 39	60, −60, 10	3.98
(c) Placebo intervention: Male group (*n* = 30), *t*‐values ≥ −3.54 indicate *p*‐values ≤ .05			
Alpha	Limbic lobe, parahippocampal gyrus, 28, 35	25, −20, −10	−3.78
Alpha	Sublobar, insula, 13	35, −15, 20	−3.54

### 3.3 Placebo intervention: Behavioral and eLORETA

As previously reported (Aichner et al., 2019; Meissner et al., 2020), the placebo‐exposed individuals showed fewer symptoms of acute nausea on day 2 than on day 1 (*day* × *group* interaction, F(1,66) = 44.83, *p* < .001; Figure 2c,e). Bonferroni‐corrected post hoc tests indicated lower levels of acute nausea on day 2 in the placebo group (*M* = 2.4, SE = 1.9) than in the control group (*M* = 4.8, SE = 1.8, *p* < .001). Neither the two‐way interaction of *day* × *sex* nor the three‐way interaction between *day* × *group* × *sex* was significant (*p*s ≥ .05).

To evaluate changes within groups, eLORETA during acute nausea on day 2 was compared to acute nausea on day 1 separately for the placebo and the no‐treatment control group. No significant effects were found in the control group (all *p* ≥ .05) or when stratified by sex (in males: all *p* ≥ .05, in females: all *p* ≥ .05). In the placebo group, no significant differences were found when the analyses were performed jointly for both sexes. When stratifying for gender, placebo‐related activation could be observed in females (extreme *p* ≤ .02, *t* = 3; Figure 2d, Table 2) and in males (extreme *p* ≤ .03, *t = −*3.78, Figure 2f, Table 2). Specifically, the female placebo group showed a decrease in alpha and beta activation from peak voxels in the parietal, temporal, and frontal lobes. In the male placebo group, there was an increase in alpha activation from peak voxels in the limbic lobe and the insula.

Sensitivity analyses were performed to test whether the EEG changes following the placebo intervention were related to the presence or absence of electrotactile stimulation of the TENS device in the two original placebo groups. For this, we separately analyzed placebo participants with (15 males and 15 females) and without (15 males and 15 females) electrotactile stimulation and compared acute nausea on day 1 and day 2 separately for both sexes by using paired *t*‐tests. No significant changes were found in the placebo group with electrotactile stimulation, neither in males nor in females. In contrast, in the female placebo group without electrotactile stimulation, eLORETA revealed activation from peak voxels in the frontal lobe in the alpha spectrum (extreme *p* ≤ .05, *t* = 4.04). No significant changes were found in the male placebo group without electrotactile stimulation.

### 3.4 Placebo effect on acute stress: Behavioral, delta power, cortisol, and amylase data

**FIGURE 3:**
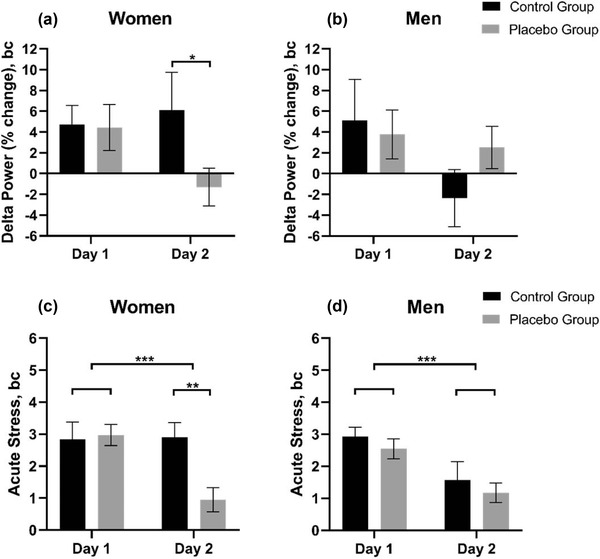
Placebo effect on delta power at C4 (top) and placebo effect on acute stress (bottom). Top: Percentage of delta power of the total fast Fourier transformation (FFT) spectrum during acute nausea (mean averaged over t = +10 to +20) in women (a) and men (b) on days 1 and 2, respectively, for the placebo (*n* = 30) and the control group (*n* = 60). The mean delta power was baseline corrected (Bc). Bottom: Baseline corrected (bc) 11‐point numeric rating scale of acute stress rated at *t* = +10 for the control (*n* = 30) and the placebo group (*n* = 60) on days 1 and 2 for (c) women and (d) men. Error bars indicate standard error. *p ≤ .05, **p ≤ .01, ***p ≤ .001

The ANOVA based on stress ratings obtained at time point +10 min on days 1 and 2 revealed a main effect of *day* (*F* (1, 74) = 30.71, *p* < .001), an interaction between *day* × *group* (*F* (1, 74) = 4.88, *p* = .03), and a three‐way interaction between *day* × *group* × *sex* (*F*(1, 74) = 6.10, *p* = .02). The three‐way interaction was due to a significant *day* × *group* interaction in women (*F*(1, 43) = 14.07, *p* < .001; Figure 3c) but not in men (*F*(1, 43) = 0, *p* > .05; Figure 3d). Bonferroni‐corrected post hoc tests indicated a placebo effect in women on day 2 (*p* = .003). In contrast, men showed a stress reduction across groups from day 1 to day 2 (*F*(1, 43) = 23.3, *p* < .001).

At C3, there was a main effect of *day* (*F*(1, 76) = 3.97, *p *= .05), although a post hoc test across groups indicated no significant difference between the nausea‐related percentage of delta power on day 1 versus day 2 (*p* ≥ .05). No other significant main effects or interactions were found for the percentage of delta power at electrode C3 (*p*s ≥ .05).

A repeated‐measures ANOVA comparing *day*, *group*, and *sex* for the percentage of delta power (0.5–4 Hz) at the C4 electrode revealed no significant effect of *day* or *day* × *group* (*p*s ≥.05), although there was a significant three‐way interaction between *day*, *group*, and *sex* (*F*(1, 76) = 4.67, *p *= .03). The interaction was driven by a significant *group* × *sex* interaction on day 2, *F*(1, 80) = 5.99, *p* = .02). Bonferroni‐corrected post hoc tests indicated a blunted increase in the percentage of delta power in the placebo group compared to controls on day 2 in women (*p* = .044; Figure 3a) but not in men (*p* > .05; Figure 3b). Sensitivity analyses were performed to test whether the placebo effect in women was related to electrotactile stimulation. When the placebo group with electrotactile stimulation was included in the ANOVA model, no significant three‐way interaction between *day*, *group*, and *sex* was found (*F*(1, 51) = 1.21, *p *> .05). In contrast, inclusion of the placebo group without electrotactile stimulation in the model revealed a significant three‐way interaction between *day*, *group*, and *sex* (*F*(1, 52) = 5.55, *p *= .02). The interaction was driven by a significant *group* × *sex* interaction on day 2, *F*(1, 52) = 7.34, *p* < .01). Bonferroni‐corrected post hoc tests confirmed a blunted increase in the percentage of delta power on day 2 in the placebo group with electrotactile stimulation compared to controls in women (*p* = .048) but not in men (*p* > .05).

For cortisol, the ANOVA resulted in a main effect of *day* (*F*(1, 85) = 6.13, *p *= .02), with an overall reduction in the levels of cortisol from day 1 (*M* = 0.15, SD = 0.76) to day 2 (*M* = −0.04, SD = 0.72). There were no main effects on amylase either for *day* or for *group* or *sex* or interactional effects (all *p*s ≥ .05; results not shown).

Among females only, the placebo effect on delta power at C4 was negatively correlated with baseline corrected stress levels at +10 min on day 2 (*r_s_* (30) = −0.41, *p* *= *.02). In addition, the placebo effect on nausea in females was negatively associated with the amylase level at acute nausea at +35 min on day 2 (*r_s_* (30) = −0.46, *p* ≤ .05).

## 4 DISCUSSION

The present study was designed to achieve a better understanding of the placebo effect in nausea by investigating its underlying neurobiological mechanisms. To this end, brain activity during a placebo intervention for anticipatory and acute nausea was studied, resulting in three main findings.

First, the placebo intervention, a combination of sham acupuncture and expectancy manipulation of nausea improvement, robustly reduced expected nausea intensity. On a cortical level, the improvement in nausea was accompanied by an increase in frontal activity and ACC activation in both sexes, which may index the placebo‐related modulation of the mindset toward a positive treatment outcome. This assumption is reasonable in light of several previous placebo studies, which have posited that the prefrontal cortex and ACC are key areas during anticipation of a beneficial effect (Benedetti, 2010; Meissner et al., 2011; Petrovic et al., 2005; Wager et al., 2004). For example, in placebo anxiolysis, which reflects the reduction of fear and anxiety following placebo treatment, changes in prefrontal activity and ACC were detected during the anticipation phase, and ACC activity was positively related to positive expectation toward the placebo treatment and the perceived placebo effect (Petrovic et al., 2005).

Second, the placebo intervention significantly alleviated acute nausea similarly in both sexes, while cortical changes differed between men and women. In male participants, the improvement of nausea in the placebo group was accompanied by an increase in alpha activation from peak voxels in the limbic lobe (parahippocampal gyrus; Brodmann area (BA) 28, 35) and the insula (BA 13). In female participants, reduced nausea was associated with decreased alpha and beta activation from peak voxels in the postcentral gyrus of the parietal lobe (BA 1, 3), in the precentral gyrus of the frontal lobe (BA 4), and in the middle temporal lobe (BA 39). The sex‐specific pattern fits well with placebo research in other areas, where sex appears to have a significant impact on the placebo effect at the physiological level but not on behavioral outcomes (Averbuch & Katzper, 2001; Casper et al., 2001; Colloca et al., 2016; Theysohn et al., 2014; Weimer et al., 2015). Activation of the primary somatosensory cortex (SI) during acute nausea was recently reported by Napadow et al. (2013), who interpreted it as a possible sign of altered afferent activity from gastric mechanoreceptors to SI during nausea‐induced tachyarrhythmia. The reduced activation of the SI (i.e., the postcentral gyrus of the parietal lobe; BA 1, 3) in our female participants thus corresponds well to the placebo effect on the gastric normo‐to‐tachy ratio, which likewise occurred in females only (Meissner et al., 2020). In contrast, the placebo effect in acute nausea in our male participants could be based primarily on a modulation of interoceptive signals in the anterior insular cortex, which has been shown to process sensations of nausea (Napadow et al., 2013).

Third, the placebo effect in nausea was associated with reduced behavioral stress, a reduction in delta power in the FFT spectrum at electrode C4, and reduced activation in PFC regions in the placebo group. The central correlates have been linked to stress in previous studies (Hall et al., 2007; McEwen et al., 2015; Nater et al., 2006). Specifically, PFC activity appears to be associated with changes in autonomic activity during nausea‐related stress (Napadow et al., 2013; Toschi et al., 2017). The results thus support the previous assumption (Colloca et al., 2016; Vambheim & Flaten, 2017) that sex‐specific differences in the physiological placebo effect in nausea are based on how stress and negative emotions are regulated. This is further supported by our parallel finding that only females showed a placebo effect on the gastric normo‐to‐tachy ratio (Meissner et al., 2020), an autonomic measure of stress and nausea (Levine, 2017).

Some limitations of the present results and interpretation need to be acknowledged. A wide range of experimental paradigms are used to induce motion sickness. Some of the results presented here may be specific for nausea induced by a virtual vection drum and thus may not translate to other types of nausea. Additionally, the spatial resolution of EEG is less reliable than that of fMRI recordings and may have failed to reveal some placebo‐relevant brain structures, including brainstem regions. Additionally, the applied placebo intervention is in fact a combination of sham acupuncture‐point stimulation and verbal suggestion of nausea improvement. It is not possible to separate these components in the present study. Furthermore, the original placebo groups with and without electrotactile stimulation were merged for the present analyses, and possible cortical effects of electrotactile stimulation may have been missed. However, the results of the sensitivity analyses confirm that the EEG changes in the female placebo group cannot be attributed to somatosensory stimulation alone. Finally, the eLORETA analyses were performed separately for the placebo and control groups, which differs from the analysis approach used for the behavioral data. Therefore, eLORETA results reflect changes in the placebo groups rather than placebo‐related cortical effects per se.

## 5 CONCLUSION

The results revealed cortical changes in participants who received a placebo intervention for nausea. In particular, the expectation of reduced nausea in the anticipation period appears to be encoded similarly in men and women, specifically via an increase in activity in the PFC and ACC. Furthermore, cortical changes during nausea differed between women (reduction in activity in parietal, frontal, and temporal regions) and men (increase in activity in limbic and sublobar regions), which might be related to sex‐specific differences in stress regulation triggered by the placebo intervention.

## CONFLICT OF INTEREST

The authors declare no conflict of interest.

## AUTHOR CONTRIBUTIONS

Karin Meissner, Anja Haile, and Matthias H. Tschoep designed the study. Karin Meissner, Anja Haile, Mallissa Watts, Simone Aichner, Verena Hoffmann, and Franziska Stahlberg performed, analyzed, and interpreted the data, and Anja Haile, Mallissa Watts, and Karin Meissner wrote the manuscript. All authors reviewed the manuscript.

### PEER REVIEW

The peer review history for this article is available at: https://publons.com/publon/10.1002/brb3.2685.

## Data Availability

The data generated and analyzed in this study are available from the corresponding author upon reasonable request.

## References

[brb32685-bib-0001] Aichner, S. , Haile, A. , Hoffmann, V. , Olliges, E. , Tschoep, M. H. , & Meissner, K. (2019). The role of tactile stimulation for expectation, perceived treatment assignment and the placebo effect in an experimental nausea paradigm. Frontiers in Neuroscience, 13, 1212. 10.3389/fnins.2019.01212 31798402 PMC6863803

[brb32685-bib-0002] Averbuch, M. , & Katzper, M. (2001). Gender and the placebo analgesic effect in acute pain. Clinical Pharmacology & Therapeutics, 70(3), 287–291. 10.1067/mcp.2001.118366 11557917

[brb32685-bib-0003] Benedetti, F. (2010). No prefrontal control, no placebo response. Pain, 148(3), 357–358. 19892467 10.1016/j.pain.2009.10.009

[brb32685-bib-0004] Benedetti, F. , Pollo, A. , Lopiano, L. , Lanotte, M. , Vighetti, S. , & Rainero, I. (2003). Conscious expectation and unconscious conditioning in analgesic, motor, and hormonal placebo/nocebo responses. Journal of Neuroscience, 23(10), 4315–4323. 12764120 10.1523/JNEUROSCI.23-10-04315.2003 PMC6741114

[brb32685-bib-0005] Casper, R. C. , Tollefson, G. D. , & Nilsson, M. E. (2001). No gender differences in placebo responses of patients with major depressive disorder. Biological Psychiatry, 49(2), 158–160. 11164762 10.1016/s0006-3223(00)00966-5

[brb32685-bib-0006] Colagiuri, B. , & Zachariae, R. (2010). Patient expectancy and post‐chemotherapy nausea: A meta‐analysis. Annals of Behavioral Medicine, 40(1), 3–14. 20387022 10.1007/s12160-010-9186-4

[brb32685-bib-0007] Colloca, L. , & Benedetti, F. (2005). Placebos and painkillers: Is mind as real as matter? Nature Reviews Neuroscience, 6(7), 545–552. 10.1038/nrn1705 15995725

[brb32685-bib-0008] Colloca, L. , Pine, D. S. , Ernst, M. , Miller, F. G. , & Grillon, C. (2016). Vasopressin boosts placebo analgesic effects in women: A randomized trial. Biological Psychiatry, 79(10), 794–802. 10.1016/j.biopsych.2015.07.019 26321018 PMC4740270

[brb32685-bib-0009] Enblom, A. , Johnsson, A. , Hammar, M. , Onelov, E. , Steineck, G. , & Borjeson, S. (2012). Acupuncture compared with placebo acupuncture in radiotherapy‐induced nausea—A randomized controlled study. Annals of Oncology, 23(5), 1353–1361. 10.1093/annonc/mdr402 21948812

[brb32685-bib-0010] Farmer, A. D. , Ban, V. F. , Coen, S. J. , Sanger, G. J. , Barker, G. J. , Gresty, M. A. , Giampietro, V. P. , Williams, S. C. , Webb, D. L. , Hellström, P. M. , Andrews, P. L. R. , & Aziz, Q. (2015). Visually induced nausea causes characteristic changes in cerebral, autonomic and endocrine function in humans. Journal of Physiology, 593(5), 1183–1196. 10.1113/jphysiol.2014.284240 25557265 PMC4358679

[brb32685-bib-0011] Gadsby, R. , Barnie‐Adshead, A. M. , & Jagger, C. (1993). A prospective study of nausea and vomiting during pregnancy. British Journal of General Practice, 43(371), 245–248. PMC1372422 8373648

[brb32685-bib-0012] Hall, M. , Thayer, J. F. , Germain, A. , Moul, D. , Vasko, R. , Puhl, M. , Miewald, J. , & Buysse, D. J. (2007). Psychological stress is associated with heightened physiological arousal during NREM sleep in primary insomnia. Behavioral Sleep Medicine, 5(3), 178–193. 17680730 10.1080/15402000701263221

[brb32685-bib-0014] Hu, S. , McChesney, K. A. , Player, K. A. , Bahl, A. M. , Buchanan, J. B. , & Scozzafava, J. E. (1999). Systematic investigation of physiological correlates of motion sickness induced by viewing an optokinetic rotating drum. Aviation Space and Environmental Medicine, 70(8), 759–765. https://www.ncbi.nlm.nih.gov/pubmed/10447048 10447048

[brb32685-bib-0015] Hunter, A. M. , Leuchter, A. F. , Morgan, M. L. , & Cook, I. A. (2006). Changes in brain function (quantitative EEG cordance) during placebo lead‐in and treatment outcomes in clinical trials for major depression. American Journal of Psychiatry, 163(8), 1426–1432. 10.1176/ajp.2006.163.8.1426 16877657

[brb32685-bib-0016] Jordan, K. , Sippel, C. , & Schmoll, H.‐J. (2007). Guidelines for antiemetic treatment of chemotherapy‐induced nausea and vomiting: Past, present, and future recommendations. The Oncologist, 12(9), 1143–1150. 17914084 10.1634/theoncologist.12-9-1143

[brb32685-bib-0017] Levine, M. E. (2017). The psychophysiology of nausea. In K. L. Koch & W. L. Hasler (Eds.), Nausea and vomiting (pp. 191–209). Springer International Publishing.

[brb32685-bib-0018] McEwen, B. S. , Bowles, N. P. , Gray, J. D. , Hill, M. N. , Hunter, R. G. , Karatsoreos, I. N. , & Nasca, C. (2015). Mechanisms of stress in the brain. Nature Neuroscience, 18(10), 1353–1363. 10.1038/nn.4086 26404710 PMC4933289

[brb32685-bib-0019] Meissner, K. , Bingel, U. , Colloca, L. , Wager, T. D. , Watson, A. , & Flaten, M. A. (2011). The placebo effect: Advances from different methodological approaches. Journal of Neuroscience, 31(45), 16117–16124. 10.1523/JNEUROSCI.4099-11.2011 22072664 PMC3242469

[brb32685-bib-0020] Meissner, K. , Lutter, D. , von Toerne, C. , Haile, A. , Woods, S. C. , Hoffmann, V. , Ohmayer, U. , Hauck, S. M. , & Tschoep, M. H. (2020). Molecular classification of the placebo effect in nausea. PLoS One, 15(9), e0238533. 10.1371/journal.pone.0238533 32966280 PMC7511022

[brb32685-bib-0021] Morrow, G. R. , Hickok, J. T. , DuBeshter, B. , & Lipshultz, S. E. (1999). Changes in clinical measures of autonomic nervous system function related to cancer chemotherapy‐induced nausea. Journal of the Autonomic Nervous System, 78(1), 57–63. 10589824 10.1016/s0165-1838(99)00053-3

[brb32685-bib-0022] Napadow, V. , Sheehan, J. D. , Kim, J. , Lacount, L. T. , Park, K. , Kaptchuk, T. J. , Rosen, B. R. , & Kuo, B. (2013). The brain circuitry underlying the temporal evolution of nausea in humans. Cerebral Cortex, 23(4), 806–813. 10.1093/cercor/bhs073 22473843 PMC3593575

[brb32685-bib-0023] Nater, U. M. , La Marca, R. , Florin, L. , Moses, A. , Langhans, W. , Koller, M. M. , & Ehlert, U. (2006). Stress‐induced changes in human salivary alpha‐amylase activity—Associations with adrenergic activity. Psychoneuroendocrinology, 31(1), 49–58. 10.1016/j.psyneuen.2005.05.010 16002223

[brb32685-bib-0024] Nichols, T. E. , & Holmes, A. P. (2002). Nonparametric permutation tests for functional neuroimaging: A primer with examples. Human Brain Mapping, 15(1), 1–25. 11747097 10.1002/hbm.1058 PMC6871862

[brb32685-bib-0025] Pascual‐Marqui, R. D. (2002). Standardized low‐resolution brain electromagnetic tomography (sLORETA): Technical details. Methods and Findings in Experimental and Clinical Pharmacology, 24, 5–12. https://www.ncbi.nlm.nih.gov/pubmed/12575463 12575463

[brb32685-bib-0026] Pascual‐Marqui, R. D. (2007). Discrete, 3D distributed, linear imaging methods of electric neuronal activity. Part 1: exact, zero error localization. arXiv preprint arXiv:0710.3341.

[brb32685-bib-0027] Petrovic, P. , Dietrich, T. , Fransson, P. , Andersson, J. , Carlsson, K. , & Ingvar, M. (2005). Placebo in emotional processing‐induced expectations of anxiety relief activate a generalized modulatory network. Neuron, 46(6), 957–969. 10.1016/j.neuron.2005.05.023 15953423

[brb32685-bib-0028] Quinn, V. F. , & Colagiuri, B. (2015). Placebo interventions for nausea: A systematic review. Annals of Behavioral Medicine, 49(3), 449–462. 10.1007/s12160-014-9670-3 25515086

[brb32685-bib-0029] Reason, J. T. , & Brand, J. J. (1975). Motion sickness. Academic press.

[brb32685-bib-0030] Sanger, G. J. , & Andrews, P. L. (2006). Treatment of nausea and vomiting: Gaps in our knowledge. Autonomic Neuroscience, 129(1–2), 3–16. 16934536 10.1016/j.autneu.2006.07.009

[brb32685-bib-0031] Singh, P. , Yoon, S. S. , & Kuo, B. (2016). Nausea: A review of pathophysiology and therapeutics. Therapeutic Advances in Gastroenterology, 9(1), 98–112. 10.1177/1756283x15618131 26770271 PMC4699282

[brb32685-bib-0032] Theysohn, N. , Schmid, J. , Icenhour, A. , Mewes, C. , Forsting, M. , Gizewski, E. , Schedlowski, M. , Elsenbruch, S. , & Benson, S. (2014). Are there sex differences in placebo analgesia during visceral pain processing? A fMRI study in healthy subjects. Neurogastroenterology & Motility, 26(12), 1743–1753. 25346054 10.1111/nmo.12454

[brb32685-bib-0033] Toschi, N. , Kim, J. , Sclocco, R. , Duggento, A. , Barbieri, R. , Kuo, B. , & Napadow, V. (2017). Motion sickness increases functional connectivity between visual motion and nausea‐associated brain regions. Autonomic Neuroscience, 202, 108–113. 28245927 10.1016/j.autneu.2016.10.003 PMC5332554

[brb32685-bib-0034] Turner, M. , & Griffin, M. J. (1999). Motion sickness in public road transport: The effect of driver, route and vehicle. Ergonomics, 42(12), 1646–1664. 10643406 10.1080/001401399184730

[brb32685-bib-0035] Vambheim, S. M. , & Flaten, M. A. (2017). A systematic review of sex differences in the placebo and the nocebo effect. Journal ofe Pain Research, 10, 1831–1839. 10.2147/JPR.S134745 PMC5548268 28831271

[brb32685-bib-0036] Wager, T. D. , Rilling, J. K. , Smith, E. E. , Sokolik, A. , Casey, K. L. , Davidson, R. J. , Kosslyn, S. M. , Rose, R. M. , & Cohen, J. D. (2004). Placebo‐induced changes in FMRI in the anticipation and experience of pain. Science, 303(5661), 1162–1167. 10.1126/science.1093065 14976306

[brb32685-bib-0037] Weimer, K. , Colloca, L. , & Enck, P. (2015). Age and sex as moderators of the placebo response‐an evaluation of systematic reviews and meta‐analyses across medicine. Gerontology, 61(2), 97–108. 25427869 10.1159/000365248 PMC4405389

[brb32685-bib-0038] Witt, C. , Meissner, K. , Pach, D. , Thiele, C. , Lüdtke, R. , Ghadiyali, Z. , Deter, H.‐C. , & Zimmermann‐Viehoff, F. (2012). Stimulation of gastric slow waves with manual acupuncture at acupuncture points ST36 and PC6—A randomized single blind controlled trial. Neurogastroenterology & Motility, 24(5), 438–445. 22309404 10.1111/j.1365-2982.2012.01877.x

